# Digital Death Humour: Exploring the Role of Humour in Death-Related Content on TikTok

**DOI:** 10.1177/00302228251327699

**Published:** 2025-03-18

**Authors:** Moa Eriksson Krutrök

**Affiliations:** 1 Department of Culture and Media Studies, 8075 Umeå University, Umeå, Sweden

**Keywords:** TikTok, humour, death, discourse, afterlife, mourning

## Abstract

This study explores the role of humour in shaping digital death discourses on TikTok. examining how users engage with mortality, the afterlife, and dying in playful yet profound ways. Through an analysis of three content strands--#celebritydeathprank, Heaven Receptionist skits, and mourning-dedicated accounts--it investigates how TikTok's participatory culture enables users to navigate and reframe death through creative and often comedic means. Rather than centering grief, this study highlights how humour serves as a mechanism for engaging with existential themes, fostering communal rememberence, and reimagining collective imaginaries of death. By leveraging TikTok's affordances--such as remixing, commenting, and algorithmic visibility--users produce content that blurs the boundaries between adversity and the absurd, intimacy and spectacle. This study contributes to research on digital death by demonstrating how social media platforms cultivate new vernaculars and ephemeralities of death discourse, where humour becomes the central mode of engagement with mortality.

Death is a sensitive subject in many regards. It hurts, it shapes us, and sometimes it is simply blatantly absurd. This work centres around the role of humour in digital death discourse as expressed on TikTok. Although humour and death may appear as contrasting themes, the convergence of these topics online reveals complex cultural, emotional, and social processes at play. With digital platforms like TikTok becoming arenas for open and inventive discourse on mortality, exploring humour in these spaces offers a fresh perspective on how death and loss becomes navigated in digital spaces. Social media platforms has increasingly become spaces where people process grief publicly and creatively. These platforms allow users to resist, reshape, and redefine traditional norms surrounding death, opening avenues for expressions of grief that are as varied and complex as the individuals posting them. Potentially, much like television can be a “medium of direct, intimate and domestic address”, these digital mediations of our mortality could potentially “bring us closer to death” (Wheatley, 2024, p. 5). In our scrolling, viewing, interacting, and understanding death as expressed on TikTok, so can our ideas of death, grief and mourning be shaped via these ephemeral digital traces. It draws users closer to their own mortality, creating new opportunities for communal expressions of grief and remembrance. These digital spaces provide a venue for mourning and engaging with death through humour—a powerful, often subversive mode of discourse that can challenge the taboos and sensitivities surrounding death.

This study builds on existing scholarship, such as existential media studies (Lagerkvist, 2022) and connective grief practices on social media (Hoffman et al., 2020), to examine how social media users employ humour to address death. Through digital ethnographic methods (Pink et al., 2016), this paper analyses three specific strands of content on TikTok: death pranks (e.g., #celebritydeathprank), afterlife skits (e.g., @taryntino21’s ‘Heaven Receptionist’ character), and grief confessionals (@sarawollner and her sisters’ interactions with their late mother’s memory). These case studies reveal how humour serves not only as a coping mechanism but as a radical social practice—one that reimagines grief, fosters communal resilience, and challenges Western society’s reluctance to engage openly with death. Rather than seeing these expressions as individual, personal, and sacred, this paper proposes death humour to be understood as a facilitator of death resistance or as a “radical move in a world that would sooner avoid the subject altogether” (Kneese, 2023, p. 193). Humour, then, can serve as a tool for individuals to express emotions, engage with societal norms, and become a vehicle for reflecting on and challenging social norms (Gray et al., 2009). Mourning norms can be questioned through the incorporation of humour, with various styles serving different purposes. In the words of Milstein, this can create brave spaces where speaking honestly about grief can become a “collective aspiration to repair the world as well as ourselves” (Milstein, 2017, p. 9).

## Mediations of Death and the Afterlife

Death becomes represented and mediated in different ways in society. Wheatley (2022) has explored television’s role in reanimating traumatic histories through spectral figures and haunting narratives. She argues that ghosts in television dramas, documentaries, and even public service announcements are not merely plot devices but conduits for confronting unresolved histories and injustices. The ghost on television functions as a reminder of the past, bringing unresolved trauma into present consciousness. Wheatley extends this idea by examining how “television itself can be ‘haunting,’” not only through narrative but also through its archival nature. Television archives, thus, become “haunted spaces” where past traumas and social wrongs are re-examined posthumously. This archival haunting, Wheatley suggests, allows television to act as a medium for societal exorcism, confronting hidden histories and facilitating a collective working through trauma and loss.

In her 2024 book, *Television/Death*, Wheatley expands on these themes, discussing how television portrays the afterlife and the societal values that shape these depictions. She examines recent comedic portrayals of the afterlife, including shows like *The Good Place*, which present distinctly American views on life after death influenced by secular and religious sources. These portrayals reflect contemporary societal concerns about life, death, and digital immortality. In this work, she highlights television’s role in helping audiences “work through” grief, positing that televised narratives of the afterlife offer ways to cope with and understand death in a culture fascinated with the dead yet constantly seeking closure. Television also provides viewers a profound connection to past lives through archival footage. By “bringing the dead back into the rooms from which they might otherwise have been absent,” Wheatley (2024: 257) argues, television provides a unique form of continuity, allowing viewers to interact with images of deceased loved ones or their own younger selves, seeking moments of connection and glimpses of a past they can revisit, if only fleetingly, through archived broadcasts. This archival aspect, Wheatley posits, makes television a site where personal histories intersect with public memory, allowing individuals to reconnect with past selves and loved ones through the communal experience of watching. Relatedly, one may wonder not only about the role of digital media for its archival qualities, allowing users to revisit the deceased, but also about the everyday ephemeralities of death when transmitted through our devices and carried around in our pockets.

## Social Media as Spaces for Mourning

Social media has emerged as a pivotal space for individuals navigating the complex terrain of grief. It serves as a sanctuary where societal norms surrounding mourning can be challenged and redefined, with various platforms facilitating sharing death-related content. Young adults, for example, harness platforms like Facebook to navigate the loss of loved ones who were integral to their online communities. Hoffman et al. (2020) underscore social media’s dual role as both a catalyst for communal remembrance and a repository for individual expressions of sorrow.

In this context, social media acts as a memorial ground for the dead. Previous research has examined how digital platforms serve as spaces for mourning and memorialisation. In this context, social media acts as a memorial ground for the dead. Within closed Facebook groups, for instance, grieving parents find solace in sharing intimate memories and photos of their departed children, protected from societal judgment (Refslund Christensen et al., 2017). This idea is echoed by Leaver (2019), who discusses how these digital sanctuaries help bereaved individuals keep their loved ones present in everyday online interactions, while Hård af Segerstad et al. (2020) examine how ongoing engagement with these digital remnants fosters enduring emotional bonds.

Building on these discussions, Sumiala and Jacobsen (2024) introduce the concept of “spectacular death,” describing how mediatisation, commercialisation, and re-ritualisation have reshaped collective imaginaries of mortality in modern society. While earlier frameworks described death as hidden or invisible, spectacular death emphasises its public visibility, primarily through media. This visibility is further extended in the realm of digital death, which involves networked, participatory forms of mourning and memorialisation enabled by platforms like TikTok. This shift—from traditional media-driven narratives to user-generated, vernacular engagements with mortality—adds a dynamic layer to how grief and death are collectively experienced and performed.

Several studies highlight the multifaceted role of social media in the grieving process across diverse populations and circumstances. Williams and Merten (2009) found that adolescents actively use social networking after the loss of a peer, directing messages to the deceased, sharing memories, and finding coping strategies through this digital space. This study reveals that online memorials allow teenagers unlimited freedom to reflect on their relationships with the deceased, suggesting that digital spaces allow for personalised grieving practices. Kasket (2012) similarly discusses the phenomenon of “continuing bonds” (Klass et al., 1996) on Facebook, emphasising how social media serves as a modern-day medium for posthumous communication and ongoing connections with the deceased. This research shows that Facebook groups allow bereaved users to address and communicate with the deceased, supporting theories that view these practices as part of a technologically mediated “continuing bonds” approach to grief.

Social media influences grieving processes beyond the scope of the individual to also reflect larger societal dynamics, not least during celebrity deaths such as Stephen Hawking (Akhther & Tetteh, 2021) or characters in television dramas (DeGroot and Leith, 2018). For example, in DeGroot and Leith’s (2018) study, they found that after the fictional death of Dr Lawrence Kutner, a character on the television series *House, M.D*, fans of the show created a Facebook memorial page that quickly became a space for expressing grief over this fictional loss. This way, fans exhibited forms of “parasocial grief” similar to those seen in real-life mourning. The study identified three main elements of parasocial grief: expressions of emotional sorrow, reminiscing about Kutner’s character, and advocacy for the character’s memory. Fans’ posts mirrored typical grieving behaviours, demonstrating deep attachments and loss often associated with real-life relationships. Notably, this grief was “disenfranchised”—a type of grief not widely recognised as valid—because the character was fictional. Nevertheless, fans used social media to mourn in ways that blended reality and fiction, revealing how online platforms can enable and intensify parasocial connections and grieving processes.

Death is commemorated in many ways using digital platforms, and often in ritualised ways. As Lagerkvist (2017) articulates, our “digital thrownness” situates us in a world where we are tasked with making meaning amidst uncertainty. Social media has become a multifaceted tool in this endeavour, supporting individuals as they navigate grief, remember loved ones, and even confront existential issues through new forms of digital expression. Cupit et al. (2021) underscore social media’s significance for college students coping with loss, suggesting the need for institutional support, highlighting how grief expressions on social media vary cross-culturally but often emphasise a shared social function in commemorative posts. Gibbs et al. (2015) point out how “funeral selfies” on Instagram serve as contemporary expressions of mourning rituals, blending personal presence with public digital interaction. Studies such as these highlight social media’s integral role in redefining grief and mourning, advocating for continued research into how humour and the sociotechnical conditions of digital platforms shape digital grief practices. The ways social media, as such, shape both grieving practices and how death is discursively shaped online make these contemporary forms of mourning practices essential for a broader understanding of death discourse.

## The Role of Humour in Mourning

Previous studies have underscored humour’s nuanced role in grief, ranging from a positive force in bereavement to a potential stressor depending on individual contexts and coping strategies. In their study of bereaved spouses, Lund et al. (2009) highlighted the significant role humour, laughter, and happiness play in aiding bereavement adjustments. The majority of participants reported both valuing and experiencing these emotions more than anticipated, with positive emotions correlating with reduced grief and depression. These findings suggest that positive emotions, including humour, can contribute to healthier bereavement outcomes, regardless of how much the individual values them, supporting the integration of positive psychology in grief support practices.

Humour can also become a central component in the social support of friends after losing a loved one. Huisman and Lemke (2022) found that humour emerged as a central element in the support dynamic and was deeply appreciated by the widows in the study, who valued its continuity from their pre-bereavement friendships into their grieving period. The study illustrated how humour became a shared, co-constructed experience, requiring a mutual understanding of “when and how” humour could be appropriately expressed. This delicate balance allowed friends to connect and support each other through laughter, respecting both the gravity of loss and the enduring bonds of friendship. Humour, when used sensitively and collaboratively, became a “risk worth taking” that enriched the grieving process, highlighting the power of relational history in shaping supportive exchanges (Huisman & Lemke, 2022, p. 57). Overall, the study suggests that humour can be an essential, nuanced component of social support after loss, emphasising that support rooted in a shared history and mutual understanding holds particular value in the grieving process.

Humour may function as a coping tool, but can also trigger grief itself. Wilson et al. (2024) found that humour was often employed to manage grief, providing moments of relief and recontextualisation. This suggests that humour is generally not recognised as a formal coping mechanism for grief; it holds the potential as a supportive measure for some bereaved individuals. However, humour’s subjective nature can be challenging within grief support since what is humorous for one person may be understood as distressing for another. Wilson et al. (2024) stress the need for more attention to bereavement humour as a possible therapeutic intervention.

Additionally, not all forms of humour have been found to aid in grieving. In their study of predictors of complicated grief and related health difficulties, Peak et al. (2024) found that humour, while generally a positive coping tool, could predict health issues when used maladaptively. Psychological research concurrently categorises humour into four dimensions: self-enhancing, affiliative, aggressive, and self-defeating (Martin et al., 2003). This way, humour can take many forms, and be complex, when integrated as part of an ongoing grieving practice. In digital spaces, self-deprecating humour is prevalent as a way of coping with daily struggles outside of a grieving context (Ask & Abidin, 2018). Here, memes and other forms of internet humour can provide a context for commiseration over shared experiences and emotional well-being. In this way, the online contexts for communicating stressors may be shaped by forms of humour that may well be maladaptive to individuals in grief. Because of this, this research focuses on digital discourses of death and the role humour plays in these digital contexts.

However, gallows humour, also known as dark humour, has been found to play a cohesive role in society, particularly in times of trauma. It has been seen as an adaptive trait, helping people navigate difficult situations (Kuiper, 2012). Orbdlik (1942) highlighted its sociological function during the Nazi occupation of Czechoslovakia in 1938–1945, noting how it fostered group cohesion and boosted morale amidst violence. Similarly, humour emerged as a coping mechanism after large-scale disasters like 9/11, aiding communal recovery (Ellis, 2003). In my previous work (Eriksson Krutrök, 2021), I examined how TikTok algorithms facilitate and shape expressions of grief. Through interactions like duets and stitches, users co-create humorous or reflective grief content that challenges traditional norms of mourning, demonstrating how algorithm-driven social media spaces shape modern grief practices and community building among those in mourning. Here, an “algorithmic closeness” in mourning was identified as a structure of content and users on TikTok, and how this aided a sense of community among them. Here, there is a need to dive deeper into the role of specific grief expressions, and especially, the role of humour when shaping a digital death discourse. 

## Methods and Material

This research is based on an ongoing digital ethnographic study (Pink et al., 2016) of the Grieftok space, which began in 2020 (Eriksson Krutrök, 2021, 2023). The case studies of this paper focus on three specific strands of content creation: one death-related TikTok challenge, one account for comedic skits and one mourner’s account. Because of this, the study centres on content from a wide range of creators and contexts across the platform to understand not only the role of dark humour in one specific form but also the complexities and disparities of different forms of content creation and specific creators. These different forms of content have been tracked, engaged with and followed from December 2022 until the autumn of 2024, where different periods of peaks and low engagement have shaped the ebbs and flows of trends and content creation regarding this study. (Table 1)

**Table 1.** Data overview.

Digital ethnography	TikTok challenge	#celebritydeathprank (and corresponding hashtags)	∼500 videos	December 2022
	Skit series	@taryntino21’s the heaven receptionist series	53 videos and comments	Oct 2023-Dec 2024
	Confessional series	@sarawollner’s (and sisters) confessions to their late mother	17 confession videos and response videos	Nov 2023-Nov 2024

Marking the first of these strands, the celebrity death prank challenge (#celebritydeathprank and corresponding hashtags) had a strong engagement on the platform through the Christmas holidays of 2022, where TikTok users pranked their families by announcing their favourite celebrities dead as they gathered for the holidays. As explained in The Rolling Stones a few days after Christmas, “TikTok was filled with shock and devastation as kids tortured their families and loved ones with erroneous celebrity deaths” (Blistein, 2022). The trend became a big hit across the platform for its crude joking material – pretending celebrities such as George Clooney, Adele, and Oprah Winfrey had suddenly died and announcing it to their distraught families – but was also criticised for being “disgusting” (Vassell, 2023). Some of the most used hashtags marking the trend (#celebritydeadprank, #celebritydiedprank, #deadcelebrityprank, #celebritydead, #deadprank) with an average of 65.2 million number of views per hashtag, indicating their reach and virality on the platform. During the period of the fieldwork, the content posted was viewed and reviewed as part of the digital ethnographic approach to “follow the data” (Sumial & Tikka, 2020) as it was continuously posted on the platform while taking screenshots and fieldnotes throughout the process of analysis. The TikTok videos analysed for the purpose of this paper were manifold: both uploaded by individual users from their family homes and compilation videos of the best versions of the trend, both reposted TikTok videos and content from other social media sites (such as Snapchat).

Second, the analysis builds on material from a user primarily creating comedic content on her TikTok page. While multifaceted in her content creation, the user @taryntino21 has gained widespread popularity and praise for her comedy series on TikTok centring around the character “The Heaven Receptionist”, in which she plays the role of a receptionist called Denise, often chewing gum, answering dumb questions over the phone, and welcomes different characters who have just entered into heaven. On her feed, she has posted 53 TikTok videos in her Heaven Receptionist series since her very first video in March 2023, which has 5.3 million, and roughly half of her videos since have been video responses to her followers’ requests, such as “would you mind meeting my sister, she died a month ago?”. Her videos have gained roughly 270.000 likes per video, becoming a viral sensation in her white bathrobe and towel turban with a disposable razor stuck into it – resembling a secretary’s headset – in front of a green screen of an image of a blue sky with scattered clouds, a classic “fluffy cloud heaven” often depicted in Christian imaginings of the afterlife (Garrett, 2015, p. 7). However, the images have watermarks on them as if downloaded in a rush when googling, connoting them somewhat as ironic rather than religious. As explained in an article in NPR, it appears “[h]eaven has a bathrobe-clad receptionist named Denise” these days, and “for the TikTokers watching along, she has become a tool for thinking through the afterlife—and for grieving those who’ve already made their way there” (Olson, 2023). At the time of writing, she has gained 1.5 million followers.

The third strand of content is a series made by a mourner on TikTok, sharing stories of the post-death life events of herself and her sisters after her mother’s passing of pancreatic cancer in 2022. As a way of dealing with her passing, and feeling isolated in her grief, Sara Wollner began making TikToks together with her sister where their wheezing laughter became an important hallmark of the series. The “Konfessions to Kare” series began with a single TikTok in November 2023, “that was all genuine humour and genuine confessions”, according to Wollner in an interview (Brant, 2024). Focusing on the absurdities of death, Wollner and her sisters shared the mishaps, awkwardness and oddities of dealing with the death of their mother. The video resonated well with TikTok users, becoming a viral sensation with 4.1 million likes and over 45 thousand comments (at the time of writing). Sometimes joined by one and sometimes two of her sisters, they have shared several videos in the series, where the format is based on confessing to their mother all of the stupid mistakes they have made since their mothers’ passing, and letting anyone and everyone in on the tea. They have shared 17 videos in total within and about the series. The content creator has made content in collaboration with brands and gained an audience over time. At the time of writing, she has gained roughly 400 thousand followers.

The study was conducted by applying digital ethnography, focusing on specific parts of an online community should also focus on non-digital practices relating to these platforms (Pink et al., 2016). This ethnographic process has been ongoing since 2020, resulting in other published research papers. In this paper, however, three specific content creators have been chosen as a particular strand of digital death discourse that integrates humour as its framework. This does not mean that humour has not been prevalent in other ways in the, sometimes called “GriefTok space”, as previously explored (Eriksson Krutrök, 2021, 2023). However, these three strands have been chosen to illustrate this form of content creation and how it relates to ideas of death and grieving in digital spaces, emphasising their own expressions and the specificities of each form.

The data which this study builds upon is manifold and diverse. Ethically, social media analysis builds upon the act of care towards communities that may be affected by our research practices. Because of this, I have dealt with the data differently depending on their originator contexts (Lindgren & Eriksson Krutrök, 2024). First, the #deathprankchallenge has been followed without downloading the data and pseudonymised in order to protect the individual users. This was done by anonymising the individuals’ usernames, personal information and faces in the figures. Second, I have chosen the TikTok accounts of two content creators who are explicitly working with content creation and branded deals and who have a high following (>300 thousand), which categorises them as ‘public figure accounts’ (Lindgren & Eriksson Krutrök, 2024; Williams et al., 2017). Because of their public persona, consent has not been sought for data analysis, and their content is included without pseudonymisation. Instead, their content creation is included as data points to understand digital death discourses on TikTok.

## Analysis

### Death Pranks as Parasocial Grief Play

In December 2022, TikTok saw a surge in videos featuring users pranking their family members during their ongoing holiday gatherings. The #celebritydeathprank videos were TikTok a challenge which involved announcing the “death” of a beloved celebrity to elicit a shocked reaction, with the prankster fabricating a breaking news-style declaration such as, “Oh my god, [celebrity’s name] dead at [real-life age].” This prank drew polarised reactions—while some viewers found humour in the responses from relatives, others saw it as insensitively capitalising on grief and shock. Some commentators expressed concern about the trend or the specific family dynamics portrayed in the clips, making the comment section a hotbed for divided opinions about appropriate conduct and pranking cultures in general. Christmas, typically a season of joy and togetherness, was here briefly interrupted by the unexpected announcement of a celebrity “death.” This temporary “rupture” highlights the significance of digital death pranks as tools for exploring societal attitudes toward grief, communal identity, and disruption of normality, as posited by Sumiala (2021), who describes death as a threat to both community and individual continuity. Ultimately, the trend captured the complex relationship between digital pranking culture, dark humour, and family dynamics when it comes to contexts of death (see Figure 1).

**Figure 1.**
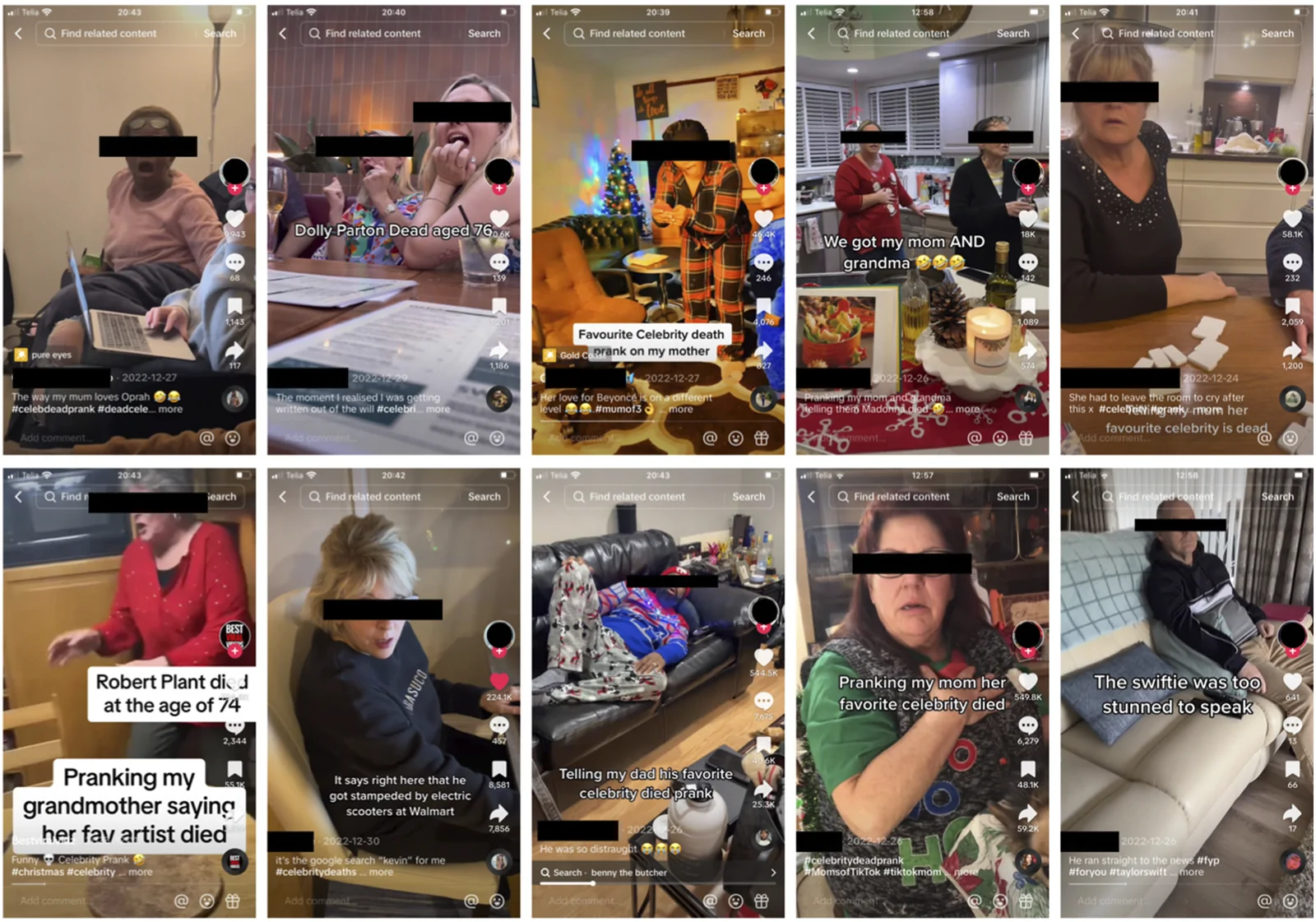
The celebrity death prank challenge.

The prank relies on the intimacy of parasocial relationships that many individuals maintain with public figures. These relationships, though one-sided, foster a sense of familiarity and emotional connection, where the figure is perceived almost like a friend or family member (DeGroot et al., 2018). When a celebrity “dies” in this prank, it disrupts this presumed connection, inviting participants to explore emotional boundaries through staged grief that allows individuals to simulate an experience of loss, “trying on” the emotional weight of mourning a public figure, though unwillingly.

The pranks were not always successful in terms of pranking. Often, the announcements were met with exclamations of disbelief and the instant grabbing of devices to google the facts. The prankers themselves often could not hold back laughter from seeing their family’s reactions, giving off the gist instantly. However, on several occasions, there were strong emotional reactions to the declarations of favourite celebrities being announced dead. Families screaming off the top of their heads, often in tandem, marked a strand of this content. In one instance, a kitchen table full of similarly dressed family members in black and red pyjamas and Christmas hats screamed as they were alerted to the statement that “Jennifer Aniston dead”, read out loud in the way news headlines usually are written. One of the adult children proclaimed, “She’s my idol! Why? How?” followed by the beginning sentence, “What the fu-” as the video ended abruptly, enhancing the video’s comedic effect. Sometimes, the reaction was less emotionally abracent and quickly turned sad. In one instance, a mother asks, “What, Kurt Russell’s dead?” followed by intense sighing “awww”, while touching her face in sadness.

The element of family dynamics in this prank adds layers of complexity. It leverages knowledge of who might be most affected by inevitable celebrity deaths, turning parasocial grief (DeGroot et al., 2018) into a performative shock. Parasocial relationships amplify these feelings of loss when confirmed celebrity deaths occur. The phenomenon of shared grief on social media following celebrity deaths has become a staple of online culture, seen in diverse responses on Twitter to Stephen Hawking’s passing, including sadness, confusion, and longing (Akhther & Tetteh, 2021). Myrick & Willoughby (2019) note that through nostalgia and shared memories, social media platforms create spaces for fans to mourn collectively. Whether for fans of Princess Diana (Turnock, 2000) or more recent figures like Kobe Bryant (Bingaman, 2022), digital mourning allows for expressions of sadness, reminiscence, and collective identity-building (Sumiala, 2021).

The #celebritydeathprank videos on TikTok offers a glimpse into this dynamic—an imitation of grief that blurs the line between the humorous and the macabre. While temporarily disruptive, these pranks underscore how parasocial relationships and the digital sphere mediate experiences of loss and collective mourning as death announcements become played out and tried on for fun. This form of play may engage creators and audiences with the physicalities of grief and their parasocial relationships—in and beyond life.

### Afterlife Skits and Digital Mourning: The Heaven Receptionist

Building on the exploration of death-related digital phenomena, this part of the analysis examines the TikTok character “The Heaven Receptionist,” portrayed by user Taryn Delanie Smith (@taryntino21) (see Figure 2). Known for her comedic approach, Smith’s character, Denise, plays the role of a sassy, gum-chewing receptionist stationed at the gates of heaven. In her videos, Denise casually fields inquiries from newly arrived souls and humorously navigating who is and is not “allowed” into heaven. The skits’ setting—a deliberately humorous take on the afterlife—has garnered immense popularity, with Smith’s portrayal resonating deeply with her audience who see her character as a comforting figure. Through humour, these skits offer audiences a space to approach death, grief, and existential questions.

**Figure 2.**
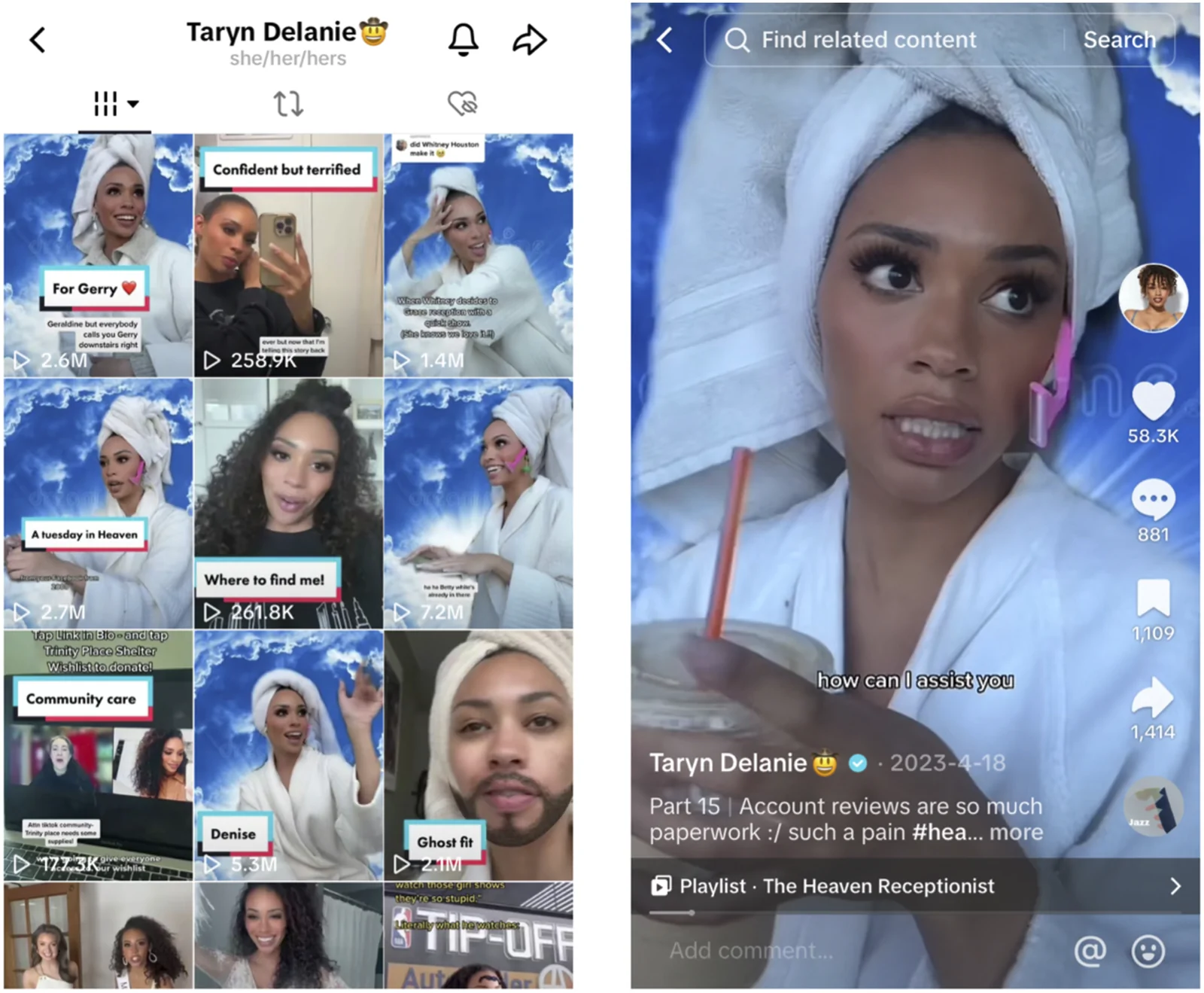
Taryn Delanie Smith (@taryntino21), a “former beauty queen, reigning chaos goblin™”, according to her TikTok bio.

Smith’s skits reveal a significant trend in digital spaces: the comedic framing of the afterlife to soften our existential anxieties about death. Her Heaven Receptionist character brings a lighthearted, almost mundane approach to the typically solemn and religious concept of the afterlife, evoking a surreal juxtaposition that appeals to many of her viewers, not unlike television series like *The Good Place.* As explored by Wheatley (2024), shows like that can function as a way to cope with and understand death. In my previous work on the role of humour and playfulness on TikTok during times of adversity (Eriksson Krutrök, 2023; Divon & Eriksson Krutrök, 2024), I have emphasised the role of play as a means of articulating vulnerabilities in ways that connect users to larger international audiences. Here, we find that with her incorporation of the afterlife and death discourse into her performance, her *playful platformed body* becomes a “medium for articulating trauma on TikTok, which, in turn, becomes algorithmically desirable” (Divon & Eriksson Krutrök, 2024).

As a former receptionist, Smith draws on her own experiences when crafting Denise, creating a character that feels both humorous and authentic – she is chewing gum, swirling her iced latte, and complaining about the broken printer. Dressed in a bathrobe and towel turban, with a razor as a makeshift headset, Denise stands out for her absurdity and relatability, creating a uniquely approachable figure for viewers navigating their feelings about death. Her skits include typical office situations combined with the occasional ghoul entering the office, clients disappearing mid-sentence because they get resuscitated and brought back to earth, and getting calls about the whereabouts of famous historical figures:

“Ra-who? Rasputin? Oh sweetheart, I don’t even have to look that up – he’s not up here! But listen, I can help locate him for you. OK? Yeah, looks like he’s someone’s sleep paralysis demon in Michigan. I can get you the name, no problem!”

Interestingly, Denise’s role as Heaven’s receptionist has taken on a life beyond its comedic intent, becoming a solace for many followers who thank her for holding space for laughter while mourning their loved ones. In this way, the Heaven Receptionist series became an interactive digital mourning space for many, as her followers express in her comments sections, similar to the ways the televised narratives of the afterlife can offer new collective coping spaces that are less focused on closure and linear ideas of grief (Wheatley, 2024). Even though she creates humorous content relating to death and the concept of an afterlife, her content seems to resonate. In fact, with roughly a thousand comments per video, it is difficult to find critical voices. Some of her followers request that Denise “meet” their loved ones at the gates of heaven, seeing her as a comforting presence for their departed relatives and friends: their departed parents, children, grandparents, etc. By leaving comments in the comments sections of her videos, these mourners make requests that Denise often meets. In one such instance, she is requested to meet the friend of a TikTok user, stating in their comment to one of her videos:

“If you don’t mind, can you welcome my friend Leah please? She’s shy and probably scared because she was only 20, and it happened so fast.”

The response was posted a few days later, and Denise met the requested individuals at the gates of heaven, always in a loving and warm manner. Utilising the “reply to comments” feature of TikTok, which allows her to create a new video with their comment included in the video, the creator meets Leah, who is presented as scared and unsure of herself, standing off-screen, as she enters the reception area of heaven. Denise hugs her and provides her with words of support:

“You were so young, I understand. But listen, if there’s something I know, it’s that there is more to being alive than just living, sometimes. ‘Cause up here you can learn and grow and play. It’s a good situation up here. You feel how warm it is? It’s like that all the time. And you can check on all the people you love, throughout their lives as much as you want to. [Pauses for Leah’s response] Yeah of course! And you can make new friends too up here if you like! Who are you a fan of? I can look em up for ya. Yeah sure! [Pauses for Leah’s response] Ok, she loves the Heavenly Meadow. I can take you there if you want. Don’t – I’m getting off anyway! [Yelling to her co-worker:] Stacey, I’m leaving early! Me and Leah are gonna go do something fun!

Arguably, her commitment to her followers to create content specifically for their needs shows that she is empathetic and a source of comfort. Smith’s ability to make followers feel seen in their grief illustrates how digital platforms can facilitate community connections around death. Smith’s skits, though comedic, provide a framework through which audiences can explore religious and existential questions about the afterlife. This blend of humour with a portrayal of death highlights the emergence of digital content that bridges the personal and the existential. Similar to what Lagerkvist (2022) describes as “existential media,” Smith’s content invites viewers to confront questions about the afterlife in a format that is accessible and relatable. Further, Wheatley’s (2024) work on television’s role in “bringing us closer to death” can extend to Smith’s TikTok skits, where the informal and direct nature of her videos aligns with television's intimacy and ability to engage viewers with death in various forms, blurring boundaries between comedy and existential contemplation.

However, at a second level of analysis, the commercialisation aspects of this practice and how this may shape the “spectacular death” (Sumiala & Jacobsen, 2024) are portrayed in her content. As a content creator, her possibilities of income, fame, and relevance on the platform are shaped by the attention gained through portraying Denise and fostering a strong connection with her viewers. As such, the incorporation of audience narratives may further show the networked natures of death in contemporary societies and its role in shaping commercialised interests within digital death discourses. The Heaven Receptionist character, nonetheless, serves as a digital space where humour, grief, and community intersect, demonstrating the evolving ways in which digital media shape our relationship with death and the afterlife. Through Denise, viewers are entertained and invited into a dialogue about mortality, transcending traditional mourning practices to create new digital rituals and forms of communal remembrance.

### Continuous Bonds and Postmortem Confessionals: Navigating Loss Through Humour

The final content strand explores the digital confessional series created by Sara Lauren and her sisters, Katie and Meghan, as they navigate life after their mother’s passing (see Figure 3). Through a series of TikTok videos, the sisters share anecdotes that they address directly to their late mother, often ending with the phrase, “We’ll continue to disappoint you.” These humorous, self-deprecating “confessions” cover their missteps in managing adult responsibilities that their mother once handled for them, such as maintaining car insurance. In one of their most popular posts, they recount how they unknowingly drove an uninsured car for nearly a year after her death. They recall their mother’s long-standing license plate, beginning with the letters AZK, saying, “RIP to AZK and whatever the numbers were—and to you,” followed by a wheezing laughter, underscoring the situation’s absurdity.

**Figure 3.**
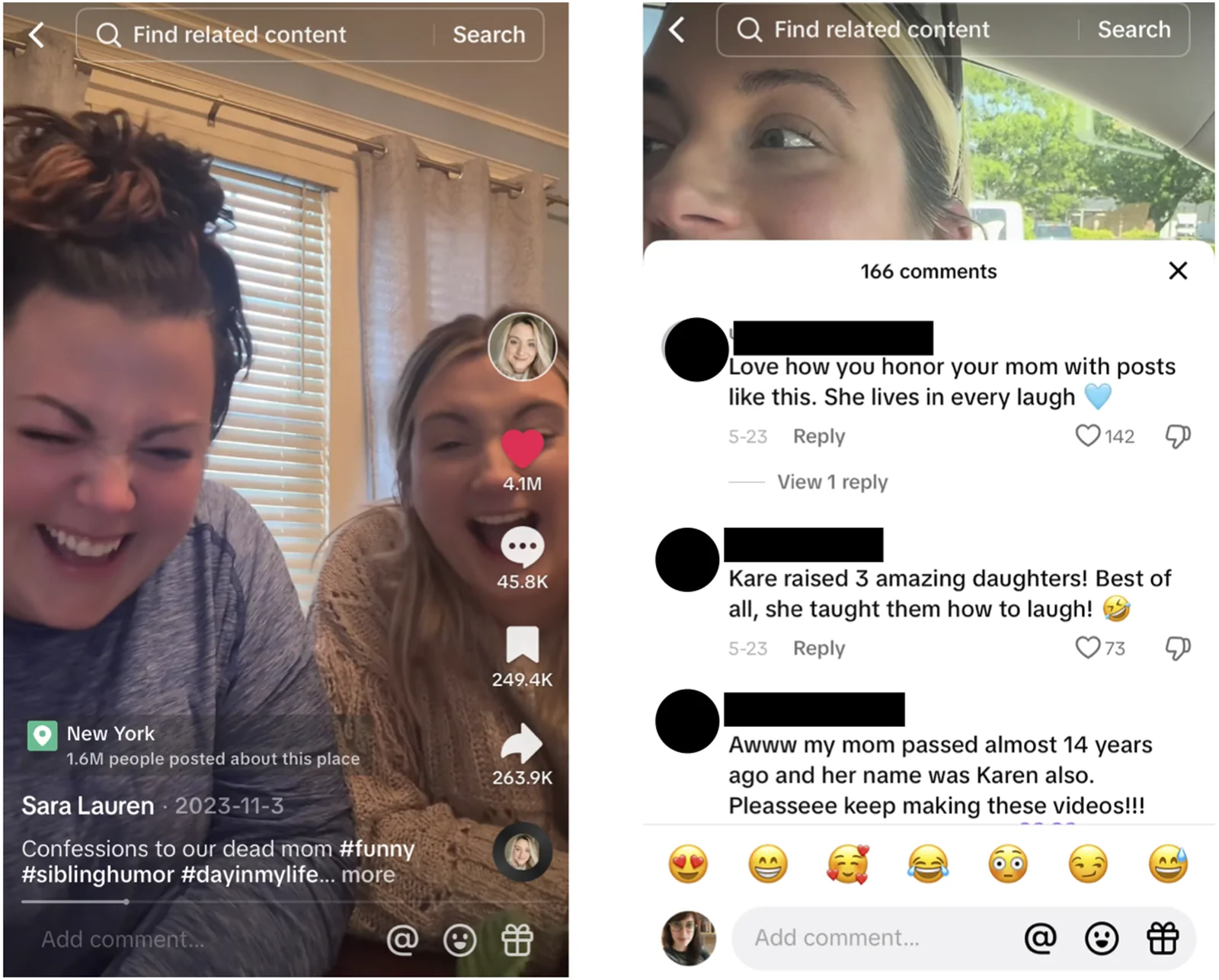
Confessions to Kare series by Sara Lauren (@sarawollner), “grieving, moving, laughing”, according to her TikTok bio.

The series has become a viral sensation by blending dark humour with their confessions. In the process, the sisters create a unique form of digital mourning that resonates deeply with audiences who see reflections of their own experiences in the absurdities and challenges of bereavement. Their approach exemplifies what Klass et al. (1996) describe as “continuing bonds,” where digital platforms allow the dead to remain active presences in our lives, also explored in digital dynamics (Kasket, 2012). By addressing their mother directly, the sisters maintain an ongoing relationship with her, turning the digital space into a site of connection and remembrance. They laugh at their foibles as they adapt to life without her guidance. The way they express their process of life after their mother’s passing, often expressed through their signature wheezing laughter, shows that digital death discourses are heavily tied to humour (see also Eriksson Krutrök, 2023). In some of these confessions, they foreclose how they have needed guidance from their bigger sister Meghan as a stand-in for their mother:

Meghan: Any time Katie thinks she might need to take a sick day, or has some kind of illness, I need to help her decide…

Katie: Should I or should I not go to work? Am I really ill? Is it in my head? Do I actually have the flu or am I just nervous? [laughter]

Sara: Nervous! [laughter] You’re so nervy! [laughter] “I’m nervous. Should I go to work?”

The sisters’ series underscores how humour can serve as a coping mechanism in the face of loss. Their self-proclaimed goal of “continuing to disappoint” their mother offers a humorous, light-hearted way to process their grief, making the often complex and bewildering aspects of postmortem responsibilities feel more manageable, echoing previous research exploring the role of humour in mourning (Lund et al., 2009; Huisman & Lemke, 2022). The series gives viewers a glimpse into how humour can facilitate resilience by reframing loss as something that, while deeply impactful, also contains elements of the absurd. In doing so, they demonstrate how mourning on platforms like TikTok can feel both communal and intensely personal.

While the grieving experience is highly personal and innate, Norbert Elias (2001 [1985]) has said it is also group-specific and shaped within collectives. Building on this point, Holmberg et al. (2019: 3) have emphasised that “[d]eath becomes real and life becomes mortal through the actions framed by the economy, media, art, scientific knowledge, law, societal institutions, and so on”. In this way, the cultural conditions of mourning are shaped by both collectives and nations, collective history and social norms. Here, death humour is shaped not only in the individual circumstance, but instead, as a communicative motive in a collective of followers and platform users that view and respond to the content. Within their comments section, they receive gratitude for talking about their late mother while still sharing their laughter. Occasionally, followers will share their own experiences of losing their parents and how this series helps them in dealing with this loss. For audiences, this confessional series brings a relatable honesty to the experience of loss, making the sisters’ story both inspirational and comforting. Their laughter and openness about the realities of adulthood highlight the often-overlooked absurdities of postmortem care, from missed paperwork to the complexities of navigating once-routine tasks. This digital mourning space allows them to celebrate their mother’s legacy while also humanising the messiness of their own lives in her absence, showing that death can be not only mourned but embraced as part of life’s strange and unpredictable continuity.

## Discussion

This study has explored the intersection of humour, grief, and digital mourning practices within an ongoing digital death discourse on TikTok. The content reveals an absurdity to parasociality in grief (DeGroot & Leith, 2018) through shocking celebrity death announcements, humour as relationality in mediated depictions of the afterlife (Wheatley, 2024) in skits, and the playfulness of continuing bonds (Klass et al., 1996) in postmortem confessions. Across the three strands examined—#celebritydeathprank, Heaven Receptionist skits, and postmortem confessions—humour acts as a shared coping mechanism, transforming grief into a more approachable, communal experience. This humour enables users to navigate their fears around mortality, creating moments of lightness and connection in digital spaces.

While previous research has shown that humour can serve as both a grief trigger and a coping mechanism (Wilson et al., 2024), humour around death on digital platforms can indeed serve as a tool for resilience, albeit with its own unique tensions. On platforms like TikTok, humour allows users to process their own grief while participating in collective expressions of mourning. This interactive, public mourning is distinct from traditional grief practices, which are often private and ritualistic. By contrast, digital spaces transform mourning into a highly performative and transient act, creating opportunities for connection but also raising questions about trivialisation and sincerity.

Digital mourning, as seen in examples like Sara Lauren and her sisters’ content, exemplifies the concept of “continuing bonds.” These bonds—whether playful, such as the sisters’ humorous portrayal of their deceased mother’s “disappointments,” or more reverent, as in requests for Denise (The Heaven Receptionist) to welcome loved ones into heaven—demonstrate how relationships with the deceased persist and adapt in new forms. Digital platforms facilitate this continuity by allowing users to maintain, project, or transform these relationships, making the dead present in a digitally mediated way. Through humour and shared interactions, such content opens new spaces for memorialisation and remembrance, reshaping how people grieve.

All three content strands engage with existential questions about death and the afterlife, offering a unique lens for understanding mortality. This reflects the broader impact of “existential media” (Lagerkvist, 2022), where life’s profound questions is adressed in accessible, often humorous formats. Platforms like TikTok offer informal, community-driven approaches to existential exploration in a world where traditional religious or philosophical frameworks may feel inaccessible. The democratising effect of this shift allows individuals and communities to grapple with mortality on their own terms, blending personal and collective understandings of death.

While certain content fosters connection and resilience, it also risks causing offence, trivialisation, and ethical dilemmas, which was especially prominent in the pranking videos. This strand sparked debates about the appropriateness of humour in grief, raising critical questions about when humour goes too far. Here, critical voices were adamant in stating the inappropriateness of pretending someone had died when they had not. In these reactions, it was more apparent that death was a topic that could not be used as fodder for pranking, at least not according to all viewers, and was not considered funny. Additionally, such practices challenge traditional boundaries of mourning, blending sincere grief with performative elements. However, trivialising death matters was not often an issue for viewers of the afterlife skits and mourning-related confessional series. Instead, by inviting others into safe spaces for mourning and laughter in combination, most commentators accentuated how vital the content was for them in their mourning of loved ones. The cultural conditions and limits of grief (Holmberg et al., 2019) can, as such, be integral to the responses where humour was not appreciated as an appropriate framing of death.

Digital spaces intersect with humour, grief, and community. Specific creators can serve as a vessel for viewers to explore mortality playfully and collectively. The Heaven Receptionist’s interactions invite audiences to imagine and engage with afterlife scenarios, creating new digital rituals and forms of communal remembrance. This blend of entertainment and existential inquiry showcases the evolving ways digital media shapes our relationships with death and the afterlife. These skits foster dialogue and connection around mortality by transcending traditional mourning practices in uniquely accessible ways. Mourning-dedicated accounts highlight death’s absurdity and humour’s role in processing grief. Through interactive content and audience engagement, these accounts create spaces where grief and humour coexist, offering new potentials for understanding loss. The platform’s affordances (Bucher & Helmond, 2018)—such as the ability to share, comment, and remix content—facilitate dynamic, communal approaches to mourning. This allows for novel expressions of grief and redefines how we relate to death in a digital context.

The findings of this study align with and extend the work of Sumiala and Jacobsen (2024), who theorised “spectacular death” as a stage where the mediatisation, commercialisation, and re-ritualisation of death have transformed collective imaginaries of mortality. Digital death, as they argue, pushes these dynamics further through the networked, participatory nature of platforms like TikTok. While spectacular death emphasises the visibility of death in public and media spaces, digital death introduces a vernacularisation of mourning, where diverse voices and everyday users contribute to collective attitudes toward death. Practices such as #celebritydeathprank or Heaven Receptionist skits exemplify how digital media democratise death engagement, allowing for profound and playful reinterpretations of mortality. This mediatised, participatory culture enables users to reimagine death in simultaneously intimate and spectacular ways, bridging personal grief with collective imaginaries. However, grief is still a risk of being spectacularised when centred on algorithmic visibility and content creation practices.

This study’s focus on TikTok and three specific types of content creation offers only a partial view of the broader landscape of digital death discourse. Future research could include other platforms and content types, exploring how different digital contexts shape mourning practices. Additionally, examining cross-cultural perspectives on digital grieving could provide deeper insights into how humour and other mechanisms function in diverse mourning traditions. Despite its limitations, this study highlights the transformative potential of digital media in reshaping how we approach grief, humour, and mortality in contemporary life. For grief professionals, this research highlights the evolving ways individuals engage with death and bereavement in digital spaces. It provides insights into how social media affects grieving processes, the accessibility of peer support, and the challenges of navigating loss in an algorithm-driven environment. Understanding these digital grief practices can help grief professionals better support individuals who integrate online spaces into their mourning rituals.
